# Clinical Results and Serum Metal Ion Concentrations following Ceramic-on-Metal Total Hip Arthroplasty at a Mean Follow-Up of 60 Months

**DOI:** 10.1155/2017/3726029

**Published:** 2017-03-08

**Authors:** W. Maurer-Ertl, D. Pranckh-Matzke, J. Friesenbichler, G. Bratschitsch, L. A. Holzer, M. Maier, A. Leithner

**Affiliations:** Department of Orthopedic Surgery, Medical University of Graz, Graz, Austria

## Abstract

*Background*. Increased metal ion levels following total hip arthroplasty (THA) with metal-on-metal bearings are a highly debated topic. Local soft tissue reactions with chronic pain and systemic side effects such as neuropathy are described. The aim of the current study was to determine the serum metal ion concentrations of Cobalt (Co) and Chrome (Cr) after THA with a ceramic-on-metal (CoM) bearing.* Patients and Methods*. Between 2008 and 2010, 20 patients underwent THA using a CoM bearing. Clinical function was evaluated by standardized scores systems (Harris Hip Score and WOMAC Score) and radiological examination included X-rays. Patient's blood samples were obtained for metal ion analysis and correlation analysis was done between these results and implant position.* Results*. Overall, 13 patients with 14 CoM devices were available for the current series. The mean age at time of surgery was 61 years (range, 41 to 85). The postoperative follow-up ranged from 49 to 68 months (mean, 58). Metal ion determination showed mean concentrations of 3,1 *µ*g/L (range, 0,3–15,2 *µ*g/L) for Co and 1,6 *µ*g/L (range, 0,1–5,5 *µ*g/L) for Cr, respectively. A correlation between cup anteversion and Co and Cr concentrations was shown.* Conclusion*. The current series showed increments for Co and Cr following CoM THA. However, these levels are lower compared to metal ion concentrations in patients with metal-on-metal bearings and the international accepted threshold for revision of MoM devices. We recommend routine follow-up including at least one obligatory evaluation of serum metal ion concentrations and an MRI once to exclude local soft tissue reactions.

## 1. Introduction

Current data from international registries are showing increasing numbers of total hip arthroplasties (THA) performed for osteoarthritis of the hip, whereas the age of the patients is decreasing [1]. Therefore, modern orthopaedic devices have to meet high demands of young and physically active patients by withstanding high stresses and strains.

Within the last decades, implant survival of THAs increased significantly by addressing complications like particle wear debris and aseptic loosening. Ultrahigh crosslinked polyethylene and ceramic-on-ceramic (CoC) bearings are known to perform very well [2, 3]. Nevertheless, hard-on-hard bearings for THA continue to be in the focus of attention, especially due to the increasing number of revision surgeries for metal-on-metal (MoM) articulations [4]. An ideal bearing surface is characterized by low wear with biological inert debris, availability of different sizes, and toleration in terms of component position [2, 3, 5, 6].

The ceramic-on-metal coupling (CoM), a ceramic femoral head articulating with a metal acetabular insert, was a novel option for hard-on-hard bearing surfaces introduced several years ago [3, 6–8]. Several in vitro analyses showed a lower risk of implant fracture, reduced wear, and metal debris, as well as squeaking phenomena compared to CoC and MoM bearings [2, 5–7, 9].

On the other hand, information on metal ion concentrations following CoM THA is limited due to the fact that there is a focus on MoM devices regarding metal wear and systemic exposure with unknown long term effects.

The aim of the current study was to analyze serum metal ion concentrations following THA with CoM bearings at a mean follow-up of 60 months. These results were compared to preoperative controls. Furthermore, clinical and radiological results were assessed. The primary hypothesis was that CoM bearings show the same performance compared to other bearings combined with low metal ion concentrations in the serum.

## 2. Materials and Methods

Twenty patients received a total hip replacement with a ceramic-on-metal (CoM) bearing between 2008 and 2010. Sixteen of these patients took part in the current study, whereas one patient received a bilateral CoM bearing, resulting in 17 devices included for the current study. Three of the 16 patients had a MoM ASR device (DePuy Orthopaedics Inc., Warsaw, IN, USA) implanted on the contralateral hip. Therefore, these patients had to be excluded for statistical analysis due to the confounding effect of a further MoM articulation.

There were seven female and six male patients with an average Body Mass Index (BMI) of 28,5 (range, 23,1–38,3). The mean age at time of operation was 61 years (range, 41–85) and the postoperative follow-up ranged from 49 to 68 months (mean: 58 months).

Indications for THA were primary osteoarthritis in 11 cases and secondary osteoarthritis due to hip dysplasia in 2 cases. Two patients had a ceramic-on-polyethylene (CoP) bearing and one further patient had a CoC bearing implanted on the contralateral side.

Preoperatively, blood was taken from 16 patients receiving a standard CoC THA and was used as an independent control for comparison with the postoperative metal ion concentrations at time of follow-up measured in the CoM patients. There were nine female and seven male patients with a mean age at time of operation of 49 years (range: 33–61). The mean BMI was 26,8 (range, 21,3–35,0).

For femoral replacement the Corail stem (DePuy Orthopaedics Inc.) was used in 15 cases and the AlloPro stem (Zimmer Inc., Warsaw, IN, USA) was used in one case. The Pinnacle cup was implanted for acetabular replacement combined with an Ultamet metal inlay (DePuy Orthopaedics Inc.). The CoM bearing was completed with a zirconia-toughened alumina femoral head (Biolox Delta, Ceramtec AG, Plochingen, Germany) with a diameter of 36 mm in all patients. Press fit fixation of components could be achieved in all cases.

The clinical and radiological follow-up included physical examination and standardized plain radiographs in two planes to determine implant position (inclination, anteversion and arc of cover). The X-rays were also reviewed for osteolysis, aseptic loosening, and implant migration. Radiological analysis was done using the Danube mediCAD Classic program (version 2.55, Hectec Gmbh; Landshut, Germany).

The Harris Hip Score (HHS) and the Western Ontario and McMaster Universities Arthritis Index (WOMAC) were used for subjective and objective clinical and functional evaluation.

Blood samples were taken by one observer (DPM) on equal setting conditions one time following an average follow-up of 58 months following index surgery. All needles and tubes were from the same batch. Serum metal ion determination was described in an earlier series in detail [10, 11]. Furthermore, the parameters for renal function and the C-reactive protein were determined and compared with the normal values from the laboratory.

The results of radiological analysis were set in correlation with the measured metal ion levels. A* p* value of <0,05 was considered to be statistically significant. For statistical analysis the PASW Statistics 16.0 program (SPSS Inc., Chicago, IL, USA) was used.

This study was approved by the Ethics Committee and written informed consent was obtained from all patients.

## 3. Results

The results of clinical and radiological assessment, as well as serum metal ion determination of 13 patients with 14 CoM articulations, are shown in Table 1.

The evaluation of functional outcome revealed a mean HHS of 81 (range, 37–99) points and a mean WOMAC score of 44 points (range, 0–136). Due to chronic pain and reduced range of motion one patient worsened the results of the used scoring systems.

**Figure 1:**
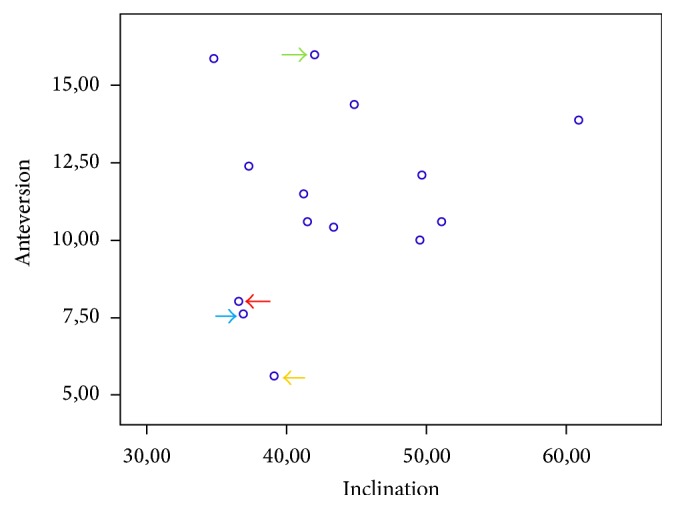
It is showing a dot plot of cup position. Patients with low inclination and low anteversion angles but high elevated Co and Cr values are indicated by the red and orange arrows. The implant positions of the patient with bilateral THA with low inclination and low anteversion angles but normal metal ion concentrations are indicated by the blue and green arrows.

Radiological evaluation did not show any case of osteolysis, aseptic loosening, or implant migration. The mean cup inclination measured was 43 degrees (range, 35–61) combined with an average anteversion of the cup of 11 degrees (range, 6–16, Figure 1). The mean calculated arc of cover was 14,3 mm (range, 9,3–16,8).

### 3.1. Metal Ions Concentrations

**Figure 2:**
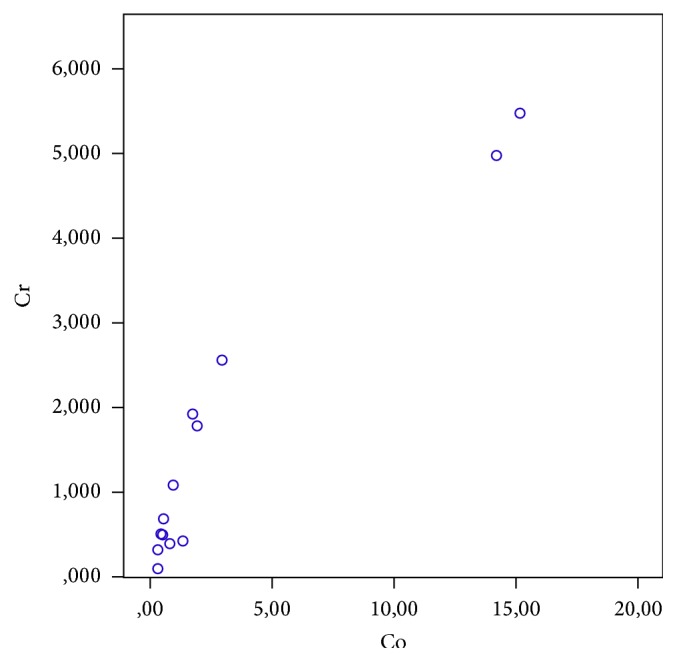
Dot plot of Co and Cr concentrations following CoM THA at a mean follow-up of 58 months.

Serum metal ion determination showed mean Co and Cr levels of 3,1 *μ*g/L (range, 0,3–15,2) and 1,6 *μ*g/L (range, 0,1–5,5) in all patients. There was one patient with bilateral CoM bearing, whereby it was shown that the levels of Co and Cr were not higher compared to patients with unilateral CoM THA. (Figure 1, green and blue arrow). Two patients showed higher increased Co and Cr values compared to the rest of the group, whereas an implant malposition might be the reason for these results (Figure 2).

Patient one had a Co concentration of 15,2 *μ*g/L and a Cr value of 5,5 *μ*g/L. Measurements showed an implant position with an inclination angle of 39 degrees, an anteversion of 6 degrees, and an arc of cover of 16,8 mm (Figure 1, orange arrow). Patient two had serum Co and Cr concentrations of 14,2 *μ*g/L and 5,0 *μ*g/L, respectively. Determination of implant position revealed an inclination angle of 37 degrees, an anteversion of 8 degrees and an arc of cover of 16,2 mm (Figure 1, red arrow).

There was one patient with bilateral CoM THA with Co and Cr concentrations of 1,9 *μ*g/L and 1,8 *μ*g/L, respectively. Determination of implant position showed an inclination of 37 degrees, an anteversion of 8 degrees, and an arc of cover of 15,8 mm for the one side (Figure 1, blue arrow). The contralateral THA showed an inclination of 42 degrees, an anteversion of 16 degrees, and an arc of cover of 16,0 mm (Figure 1, green arrow). The reason for the different implant positions was two different surgeons. The elevated metal ion concentrations were seen in patient older than 80 years at time of surgery with slightly impaired renal function which might be the reason for the observed increment.

**Figure 3:**
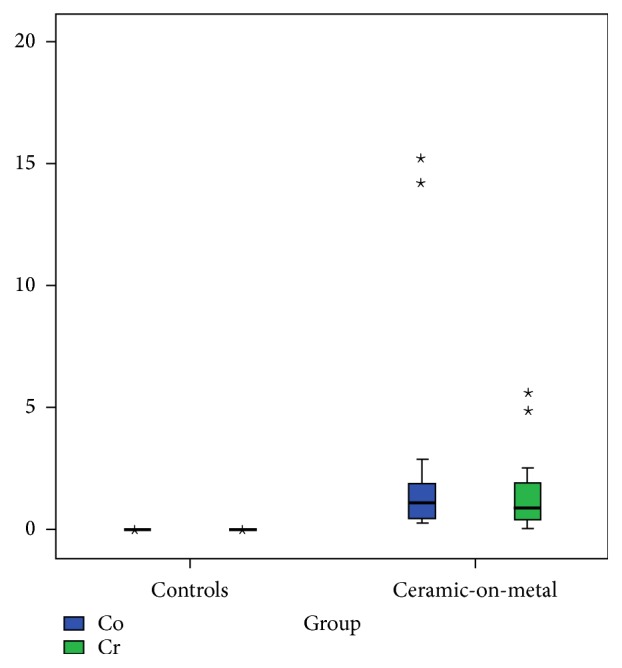
Box plot showing the serum metal ion concentrations measured in the preoperative population and following CoM THA. The Co and Cr levels were significantly higher in the CoM THA group (*p* < 0.001). ⋆ refers to the two patients with highly increased Co and Cr values in the CoM THA group.

Compared to the preoperative controls, the measured Co and Cr levels were significantly increased in the postoperative group (Co & Cr, *p* < 0.001, Figure 3). Nevertheless, the mean and the median Co and Cr levels were below the international accepted threshold of 7,0 *μ*g/L for revision surgery recommended by the UK Medicines and Healthcare products Regulatory Agency (MHRA) [21].

Pearson correlation coefficient was calculated for serum metal ion concentrations and implant position. A significant correlation could be found between Co and Cr levels and the anteversion (Co: *p* = 0.008, Cr: *p* = 0.007). No correlation could be found between Co and Cr levels and the BMI.

### 3.2. Revisions

One revision surgery had to be done in a female patient due to metal wear and chronic pain four years following index surgery. Serum metal ion concentrations were within the limits, whereas Co and Cr levels were highly elevated in the aspiration fluid. Radiologically, the inclination, anteversion, and the arc of cover were within a normal range. The renal function was not impaired. Serum Cr decreased from 1,2 *μ*g/L to 0,1 *μ*g/L from January to October 2014 postoperatively, although the Co level remained unaltered at 0,3 *μ*g/L.

## 4. Discussion

The aim of the study was to determine the serum Co and Cr levels in patients with CoM THA. Further, correlation analyses were performed for metal ion levels and component alignment parameters. Overall, two outliers were detected with elevated Co and Cr levels higher than the international accepted threshold of 7,0 *μ*g/L, whereas the implant position and impaired renal function might be the reason for this observation. Compared to the preoperative population, the Co and Cr levels were significantly elevated in CoM THA patients (*p* < 0.001).

Statistical analysis showed a correlation between acetabular anteversion and the measured Co and Cr levels. A correlation between inclination, arc of cover, serum metal ion levels, and BMI could not be shown.

Yi et al. [7] and Hill et al. [3] recently reported their results determining metal ion concentrations following CoM THA with a mean follow-up of 50 and 34 months, respectively (Table 2). Like in the current series, the serum levels of Co and Cr were significantly elevated compared to normal values without stabilization or downward trend after a running in period of the tribological bearing. Furthermore, Yi et al. [7] reported a significant correlation between metal ion concentrations and the BMI. Kazi et al. [19] and Sawalha et al. [22] also related a correlation between metal ion levels and the BMI for MoM bearings but not for CoM articulations. In the current series, there was no correlation between Co and Cr and the BMI, whereas the measured Co and Cr concentrations were comparable to the results of Yi et al. [7].

The mean levels of Co and Cr following CoM THA were significantly elevated compared to the preoperative population and the reference values of the laboratory but lower than the threshold value of 7,0 *μ*g/L given by The United Kingdom Medicines and Healthcare products Regulatory Agency (MHRA, Table 1) [21]. Nonetheless, there was a correlation between acetabular anteversion and metal ion concentrations (Table 2).

Williams et al. [23] showed lower metal ion concentrations after CoM THA compared to MoM bearings 6 months following implantation. Nevertheless, in comparison to the current series, the mean Co and Cr concentrations were higher, especially after exclusion of the patients with the MoM ASR devices.

Isaac et al. [20] showed detectable Co and Cr levels 12 months following implantation of CoM bearings, but the concentrations were lower compared to MoM devices. On the other hand, Schouten et al. [17] related no differences comparing Co and Cr levels following CoM and MoM at 6 and 12 months of follow-up (Table 2).

Kazi et al. [19] reported higher Co and Cr levels in patients with bilateral THA, which could not be proven in the current series due to the fact that there was only one patient included with normal Co and Cr concentrations.

In 2012, Maurer-Ertl et al. [11] published their results determining Co and Cr concentrations following MoM THA with the ASR devices. Comparing the mean levels of Co and Cr in the serum of patients with CoM and MoM bearings resulted in significant lower concentrations in the CoM group. The mean Co and Cr levels in the MoM group amounted to 6,0 *μ*g/L and 6,02 *μ*g/L, respectively, whereas the mean Co and Cr levels were 3,15 *μ*g/L and 1,59 *μ*g/L of the CoM group, respectively.

Hart et al. [24] showed that metal ion levels are higher in patients with MoM devices and an inclination angle higher than 50 degrees. On the other hand, three patients with an inclination angle higher than 50 degrees in the current series showed Co and Cr levels within a normal range. Lainiala et al. [4] also reported an increased acetabular inclination angle as an independent risk factor for increased metal ion concentrations in case of MoM resurfacing hip arthroplasty. Further, young age, higher range of motion, smaller femoral head size, and female sex were negative predictive factors for higher metal ion concentrations.

Yi et al. [7] related that there was no correlation between stem type, head size, and metal ion levels. On the other hand, in the study of Lainiala et al. [4] there was a significant correlation between increased Co and Cr concentrations and female sex, bigger femoral head size, higher range of motion and time of follow-up between implantation of large diameter MoM devices, and serum metal ion determination.

Another well-known source of metal ions is the taper junction between stem and femoral head, whereas, the taper-corrosional effect is lower in ceramic-on-metal taper junctions compared to metal-on-metal taper junctions [25]. Lainiala et al. [4] noticed lower metal ion levels when using a titanium (Ti) sleeve between the head and the stem resulting in a Ti-Ti interface.

Engh et al. [5] reported no differences in revision rates at 2 years between CoM and MoM THA, whereas the CoM group had no wear related revisions with low metal ion levels. Furthermore, metal ion analysis showed that Co and Cr levels in patients with CoM and MoM bearings were not different at 5 years of follow-up.

Within the next few years, an increment of bearing-related revisions will be expected, especially in case of MoM THA. Elevated serum metal ion levels and adverse reactions to metal debris (ARMD) will be the main reasons for revision which has been shown in the recent literature [16, 26, 27]. CoM bearings have to be monitored for the same type of failures on the long-run [5].

One limitation of the current study was that the serum metal ion determination was done only once at a follow-up of nearly 5 years. Therefore, it is not possible to make any statement about the course of serum metal ion concentrations.

One further limitation was the small number of patients enrolled in this series. One reason for that was the fact that the demand on the metal inlay declined resulting in a low number of implantations and the usage of the inlay depended on the surgeon's preference, additionally.

## 5. Conclusion

The current series showed increased serum metal ion concentrations following CoM THA, whereas these levels were lower compared to the threshold for revision surgery given by the MHRA. Furthermore, the Co and Cr concentrations were lower compared to the values measured in patients with MoM bearings, which is shown in the literature.

Regular follow-up with serum metal ion level determination is recommended to prevent another catastrophic outcome with high revision rates like in case of MoM devices. Magnetic resonance imaging should be done once to exclude ARMD. In case of increased serum metal ion concentrations, local pain, and/or appearance of an ARMD, revision of the tribological bearing has to be considered.

## Figures and Tables

**Table 1:** Results of clinical and radiological assessment, as well as serum metal ion determination.

	mean	median	min	max
CoM (*n* = 13 patients)				
Co (*μ*g/L)	3,1	1,1	0,3	15,2
Cr (*μ*g/L)	1,6	0,9	0,1	5,5
Creatinine (*μ*g/dL)	0,9	0,9	0,5	1,7
CRP (mg/dL)	4,6	3,5	0,7	19,5
Cup inclination (°)	43	42	35	61
Cup anteversion (°)	11	11	6	16
Arc of cover (mm)	14,3	14,7	9,3	16,8
HHS	81	88	37	99
WOMAC	44	23	0	136
Controls (*n* = 16 patients)				
Co (*μ*g/L)	0,01	0,00	0,00	0,03
Cr (*μ*g/L)	0,03	0,02	0,01	0,05

**Table 2:** Metal ion concentrations, revision rates, and time of follow-up of several studies reporting their results following MoM and CoM THA.

Study	Revision rate (%)	Metal ion concentrations (*μ*g/L)		Follow-up (months)
		Co	Cr	
Large diameter MoM				
Hug et al. [12]	13	14 (0–150)	5 (0–87)	36 (12–61)
Langton et al. [13]	6	3.26 (1.1–32)	3.71 (2.4–22)	41 (10–57)
Lavigne et al. [14]	0	1.78 (0.32–7.59)	1.78 (0.24–6.20)	24
Reito et al. [15]	36	Unilateral: 4.2 (0.3–191.7) Bilateral: 13 (1.5–139.9)	Unilateral: 2.1 (0.4–115) Bilateral: 3.4 (0.8–61)	—
Maurer-Ertl et al. [16]	32	20.1 (0.3–190.5)	12.8 (1.0–89.8)	78 (20–98)
Engh et al. [5]	3	1.01	0.95	60
Schouten et al. [17]	—	1.57	1.73	12
Lainiala et al. [4]	—	3.3 (0.3–191.7)	1.9 (0.4–114.8)	3.4 years (0.6–6.5)
MoM resurfacing				
Hug et al. [12]	12	12 (0–126)	7 (0–60)	54 (12–74)
Langton et al. [13]	3.2	2.74 (0.4–271)	4.16 (1.5–69.8)	35 (8–57)
Langton et al. [18]	1.3	1.89 (0.4–228.0)	3.61 (0.6–115.0)	26 (13–44)
Reito et al. [15]	30	Unilateral: 2.3 (0.7–217.7) Bilateral: 2.4 (0.9–96.9)	Unilateral: 2.0 (0.8–94) Bilateral: 2.7 (1–54)	—
Maurer-Ertl et al. [16]	30	16.0 (0–171.8)	13.1 (0.31–125.0)	86 (68–109)
Lainiala et al. [4]	—	1.3 (0.5–224.7)	1.6 (6.4–13.1)	4 years (1–6.8)
CoM THA				
Engh et al. [5]	1.5	0.85	1.13	60
Schouten et al. [17]	—	1.77	1.84	12
Hill et al. [3]	3.1	0.83 (0.24–27.56)	0.78 (0.21–8.84)	34 (23–45)
Yi et al. [7]	0	2.82 (±1.94)	2.41 (±1.41)	50
Kazi et al. [19]	—	1.37 (0.12–10.68)	1.09 (0.00–5.51)	24
Isaac et al. [20]	—	0.72	0.43	12
Cadossi et al. [6]	—	0.78 (0.14–1.67)	0.97 (0.11–2.61)	36
Joyce et al. [8]	10.7	—	—	18
Current series	7	3.1 (0.3–15.2)	1.6 (0.1–5.5)	58 (49–68)
